# Supplementary material for: Intragenic viral silencer element regulates HTLV-1 latency via RUNX complex recruitment
Source: Nat Microbiol. 2025 May 13;10(6):1447–62. doi: 10.1038/s41564-025-02006-7 (PMC12137137; doi:10.1038/s41564-025-02006-7)
Supplement: Supplementary file 1 — Reporting Summary [file 41564_2025_2006_MOESM1_ESM.pdf]

nature portfolio

Corresponding author(s): Yorifumi Satou

Last updated by author(s): 21/03/2025

Reporting Summary

Nature Portfolio wishes to improve the reproducibility of the work that we publish. This form provides structure for consistency and transparency in reporting. For further information on Nature Portfolio policies, see our Editorial Policies and the Editorial Policy Checklist.

Statistics

For all statistical analyses, confirm that the following items are present in the figure legend, table legend, main text, or Methods section.

n/a	Confirmed	
☐	☒	The exact sample size (*n*) for each experimental group/condition, given as a discrete number and unit of measurement
☐	☒	A statement on whether measurements were taken from distinct samples or whether the same sample was measured repeatedly
☐	☒	The statistical test(s) used AND whether they are one- or two-sided<br>*Only common tests should be described solely by name; describe more complex techniques in the Methods section.*
☒	☐	A description of all covariates tested
☒	☐	A description of any assumptions or corrections, such as tests of normality and adjustment for multiple comparisons
☐	☒	A full description of the statistical parameters including central tendency (e.g. means) or other basic estimates (e.g. regression coefficient) AND variation (e.g. standard deviation) or associated estimates of uncertainty (e.g. confidence intervals)
☐	☒	For null hypothesis testing, the test statistic (e.g. *F*, *t*, *r*) with confidence intervals, effect sizes, degrees of freedom and *P* value noted<br>*Give P values as exact values whenever suitable.*
☒	☐	For Bayesian analysis, information on the choice of priors and Markov chain Monte Carlo settings
☒	☐	For hierarchical and complex designs, identification of the appropriate level for tests and full reporting of outcomes
☒	☐	Estimates of effect sizes (e.g. Cohen's *d*, Pearson's *r*), indicating how they were calculated

Our web collection on statistics for biologists contains articles on many of the points above.

Software and code

Policy information about availability of computer code

Data collection	ATAC-seq:<br>ATAC libraries were sequenced on Illumina NextSeq 550 to obtain paired-end reads around 20 million reads using the following read length: read1_37 bp; read2_37 bp.<br>ChIP-seq:<br>ChIP DNA libraries after DNA capture were sequenced on Illumina MiSeq to obtain paired-end reads around 1 million reads using the following read length: read1_75 bp; read2_75 bp.<br>Sequencing for Cut and Run:<br>Cut and Run DNA libraries with DNA capture were sequenced on Illumina MiSeq to obtain paired-end reads around 1 million reads using the following read length: read1_75 bp; read2_75 bp.
Data analysis	TFBIND – The prediction tool of transcription factors binding site (https://tfbind.hgc.jp/)<br>GraphPad Prism 7 – The validation of Statistical significance<br>FlowJo (version 9.9.6) – FACS analysis<br>FastQC (version 0.10.0) – Checking quality of fastq file (https://www.bioinformatics.babraham.ac.uk/projects/fastqc/)<br>cutadapt (version 1.18) – Removing the adapter sequences (https://cutadapt.readthedocs.io/en/stable/index.html)<br>PRINSEQ (version 0.20.4) – Cleaning the sequence data (http://prinseq.sourceforge.net/)<br>BWA (version 0.7.12) – Alignment for DNA-seq, MNase-seq, ChIP-seq (https://sourceforge.net/projects/bio-bwa)<br>SAMTools (version 1.11) – Operating sam and bam files (http://www.htslib.org/download/)<br>picard (version 2.0.1) – PCR replicate removal (https://broadinstitute.github.io/picard/)<br>IGV (version 2.8.0) – Used to visualize mRNA-seq, MNase-seq and ChIP-seq data (https://software.broadinstitute.org/software/igv/download)

Codes used for ChIP-seq and ATAC-seq data analysis have been deposited to Github (https://github.com/satoulab/Enhancer_HTLV_NatComm/tree/main/SCRIPTS/ChIP-seq). Codes for analyzing integration site for the wt39 and wt 51 are also available in Github (https://github.com/satoulab/Enhancer_HTLV_NatComm/tree/main/SCRIPTS/DNA-seq_including_IS_analysis).

For manuscripts utilizing custom algorithms or software that are central to the research but not yet described in published literature, software must be made available to editors and reviewers. We strongly encourage code deposition in a community repository (e.g. GitHub). See the Nature Portfolio guidelines for submitting code & software for further information.

Data

Policy information about availability of data

All manuscripts must include a data availability statement. This statement should provide the following information, where applicable:

- Accession codes, unique identifiers, or web links for publicly available datasets
- A description of any restrictions on data availability
- For clinical datasets or third party data, please ensure that the statement adheres to our policy

Data supporting the findings reported in this study are available from the corresponding author upon request. Raw sequence files (fastq) for ATAC-seq, ChIP-seq and genomic DNA have been deposited to SRA under the bioproject: PRJNA1236037.

Research involving human participants, their data, or biological material

Policy information about studies with human participants or human data. See also policy information about sex, gender (identity/presentation), and sexual orientation and race, ethnicity and racism.

Reporting on sex and gender	This information is provided in Supplemental Table 1, Supplemental Table 2 and Supplemental Table 6.
Reporting on race, ethnicity, or other socially relevant groupings	This manuscript did not include the research for race, ethnicity or other socially constructed categories.
Population characteristics	ACs and smoldering and chronic ATL patients were male and female, ages 42-78(Median:67). Each sample has high PVL 2.4-35.8(Median:14.5).
Recruitment	ATL samples were obtained from patients with high PVL. Some patients supplied the samples of different time-points. We recruited all participants as volunteers without any biases and written informed consent was obtained from each participant.
Ethics oversight	This study was approved by the Kumamoto University Institutional Review Board (approval number 248 and 263) and carried out in accordance with the guidelines proposed in the Declaration of Helsinki. Informed written consent was obtained from all subjects in this study.

Note that full information on the approval of the study protocol must also be provided in the manuscript.

Field-specific reporting

Please select the one below that is the best fit for your research. If you are not sure, read the appropriate sections before making your selection.

☒ Life sciences ☐ Behavioural & social sciences ☐ Ecological, evolutionary & environmental sciences

For a reference copy of the document with all sections, see nature.com/documents/nr-reporting-summary-flat.pdf

Life sciences study design

All studies must disclose on these points even when the disclosure is negative.

Sample size	Sample sizes were chosen to provide sufficient confidence to validate the tendency of chromatin-openess, the viral and host transcription level, and TF binding in HTLV-1 infected cell lines and patient samples. We did not do any calculation for sample size.<br>We analyzed as many samples as possible in terms of clinical sample and research budget availability.
Data exclusions	No data were excluded from the manuscript.
Replication	All results shown in the manuscript were validated by duplicates or triplicates
Randomization	Randomization is not required for this study.
Blinding	Blinding is not required for this study.

Reporting for specific materials, systems and methods

We require information from authors about some types of materials, experimental systems and methods used in many studies. Here, indicate whether each material, system or method listed is relevant to your study. If you are not sure if a list item applies to your research, read the appropriate section before selecting a response.

Materials & experimental systems

n/a	Involved in the study
☐	☒ Antibodies
☐	☒ Eukaryotic cell lines
☒	☐ Palaeontology and archaeology
☒	☐ Animals and other organisms
☒	☐ Clinical data
☒	☐ Dual use research of concern
☒	☐ Plants

Methods

n/a	Involved in the study
☐	☒ ChIP-seq
☐	☒ Flow cytometry
☒	☐ MRI-based neuroimaging

Antibodies

Antibodies used

anti-RUNX1 (abcam ab23980), anti-CBFb (abcam ab195411), anti-GATA3 (CST 5852P), anti-HDAC3 (Invitrogen PA5-85378), anti-ETS1 (CST 14069S) and anti Sin3A (CST 7691); 1:250 dilution for conventional ChIP. For Cut and Run, the antibodies are used in 1:50 dilution.
FITC-labeled anti-Tax mAb (Lt4: kindly gifted by Yuetsu Tanaka (University of the Ryukyus)
mouse anti-RUNX1 (A-2; Santa Cruz Biotechnology); 1:2000 dilution
Rabbit anti-beta actin (13E5; Cell Signaling Technology); 1:5000 dilution
horseradish peroxidase (HRP)-conjugated secondary antibodies donkey anti-rabbit IgG-HRP (Jackson ImmunoResearch) ;1:10000 dilution
horseradish peroxidase (HRP)-conjugated secondary antibodies donkey donkey anti-mouse IgG-HRP (Jackson ImmunoResearch);1:10000 dilution
PE-labeled anti-RUNX1 antibody (RXDMC, Invitrogen) ; 1:20 dilution
PE-labeled anti-NGFR mAb (ME20.4, BioLegend) ; 1:30 dilution
FITC-labeled anti-HIV p24 mAb (Beckman);1:500 dilution
biotin-labeled anti CD8 mAb (RPA-T8; Biolegend);1:30 dilution
biotin-labeled CD45RA mAb (HI100; Biolegend) ;1:30 dilution
PerCP/Cy5.5-labeled anti-CD4 mAb (OKT4, BioLegend);1:30 dilution
anti-H3K4me3; 1:250 dilution
anti-H3K9Ac; 1:250 dilution
PE-labeled anti-EGFR mAb (AY13, BioLegend); 1:30 dilution
BV510-labeled anti CD8 mAb (RPA-T8; Biolegend) 1:30 dilution
APC-labeled anti EGFR mAb (AY13; Biolegend) 1:30 dilution
anti CADM1 mAb (3E1; MBL);1:1000 dilution
Alexa Fluor647-labeled Goat Anti-Chicken IgY H&L ab(Abcam);1:2000 dilution
BV421-labeled anti CD7 mAb (M-T701; BD Biosciences) 1:30 dilution
APC-labeled anti CD45RO mAb (UCHL1; Biolegend) 1:30 dilution

Validation

Further validation report could be found on the the supplier website.
anti-RUNX1 (abcam ab23980): https://www.abcam.co.jp/products/primary-antibodies/runx1--aml1-antibody-ab23980.html
anti-CBFb (abcam ab195411):https://www.abcam.co.jp/products/primary-antibodies/cbfb-antibody-chip-grade-ab195411.html
anti-GATA3 (CST 5852P):https://www.cellsignal.jp/products/primary-antibodies/gata-3-d13c9-xp-rabbit-mab/5852
anti-HDAC3 (Invitrogen PA5-85378):https://www.thermofisher.com/antibody/product/HDAC3-Antibody-Polyclonal/PA5-85378
anti-ETS1 (CST 14069S) :https://www.cellsignal.jp/products/primary-antibodies/ets-1-d8o8a-rabbit-mab/14069
Sin3A (CST 7691):https://www.cellsignal.jp/products/primary-antibodies/sin3a-d1b7-rabbit-mab/7691
mouse anti-RUNX1 (A-2; Santa Cruz Biotechnology):https://www.scbt.com/p/runx1-antibody-a-2
Rabbit anti-beta actin (13E5; Cell Signaling Technology):https://www.cellsignal.jp/products/primary-antibodies/b-actin-13e5-rabbit-mab/4970
horseradish peroxidase (HRP)-conjugated secondary antibodies donkey anti-rabbit IgG-HRP (Jackson ImmunoResearch):https://www.jacksonimmuno.com/catalog/products/711-035-152
horseradish peroxidase (HRP)-conjugated secondary antibodies donkey donkey anti-mouse IgG-HRP (Jackson ImmunoResearch):https://www.jacksonimmuno.com/catalog/products/715-035-150
PE-labeled anti-RUNX1 antibody (RXDMC, Invitrogen) :https://www.thermofisher.com/antibody/product/RUNX1-Antibody-clone-RXDMC-Monoclonal/12-9816-80
PE-labeled anti-NGFR mAb (ME20.4, BioLegend) :https://www.biolegend.com/ja-jp/products/pe-anti-human-cd271-ngfr-antibody-6428?GroupID=GROUP28
FITC-labeled anti-HIV p24 mAb (Beckman):https://www.beckman.com/reagents/coulter-flow-cytometry/antibodies-and-kits/single-color-antibodies/hiv-1-core-antigen/6604665
biotin-labeled anti CD8 mAb (RPA-T8; Biolegend):https://www.biolegend.com/ja-jp/clone-search/biotin-anti-human-cd8a-antibody-833?GroupID=BLG5903
biotin-labeled CD45RA mAb (HI100; Biolegend) :https://www.biolegend.com/ja-jp/products/biotin-anti-human-cd45ra-antibody-685
PerCP/Cy5.5-labeled anti-CD4 mAb (OKT4, BioLegend):https://www.biolegend.com/ja-jp/products/percp-cyanine5-5-anti-human-cd4-antibody-5011
anti-H3K4me3; https://www.cellsignal.jp/products/primary-antibodies/tri-methyl-histone-h3-lys4-c42d8-rabbit-mab/9751
anti-H3K9Ac; https://www.merckmillipore.com/JP/ja/product/Anti-acetyl-Histone-H3-Lys9-Antibody,MM_NF-06-942
PE-labeled anti-EGFR mAb (AY13, BioLegend); https://www.biolegend.com/ja-jp/products/pe-anti-human-egfr-antibody-7432
BV510-labeled anti CD8 mAb (RPA-T8; Biolegend);https://www.biolegend.com/ja-jp/products/brilliant-violet-510-anti-human-cd8a-

antibody-8000
APC-labeled anti EGFR mAb (AY13; Biolegend) https://www.biolegend.com/ja-jp/products/apc-anti-human-egfr-antibody-7714
anti CADM1 mAb (3E1; MBL) https://ruo.mbl.co.jp/bio/dtl/A/index.html?pcd=CM004-3
Alexa Fluor647-labeled Goat Anti-Chicken IgY H&L ab(Abcam) https://www.abcam.co.jp/products/secondary-antibodies/goat-chicken-igy-hl-alexa-fluor-647-ab150171.html
BV421-labeled anti CD7 mAb (M-T701; BD Biosciences); https://www.bdbiosciences.com/en-dk/products/reagents/flow-cytometry-reagents/research-reagents/single-color-antibodies-ruo/bv421-mouse-anti-human-cd7.562635
APC-labeled anti CD45RO mAb (UCHL1; Biolegend);https://www.biolegend.com/ja-jp/products/apc-anti-human-cd45ro-antibody-856

Eukaryotic cell lines

Policy information about cell lines and Sex and Gender in Research

Cell line source(s)

Jurkat cells were obtained from ATCC (TIB-152).
293T cells were obtained from ATCC (CRL-3216).
Molt4 cells were obtained from ATCC (CRL-1582).
Kit225(+) cells were obtained from ATCC (CRL-1990).
293T cells were obtained from ATCC (CRL-3216).
PG13 cells were obtained from ATCC (CRL-10686).
HeLa cells were obtained from ATCC (CCL-2).
K562 cells were obtained from ATCC (CCL-243).
MoT cells were obtained from ATCC (CRL-8066)
Si-2 cells were obtained from Japanese Collection of Research Bioresources Cell Bank (JCRB1321)
FLK-BLV was kindly provided by Yoko Aida (The University of Tokyo)
Plate-GP cells were obtained from Takara Bio Inc.
BCBL1, BC-2, KMS-12-PE and YT-1 cell lines were kindly gifted by Seiji Okada (Kumamoto university).
iPS-ML cells were kindly gifted by Shinya Suzu (Kumamoto university).
CEM and THP1 cell lines were obtained from Hiroaki Takeuchi (Tokyo Medical and Dental University).
MT1 cells were obtained from Dr. Michiyuki Maeda (Maeda M. et al., J Exp Med. 1985).
TBX-4B cells were obtained from Prof. Charles R.M. Bangham (Cook L.B. et al., Blood. 2014).
JET cells were obtained from Prof. Jun-ichi Fujisawa (Furuta R. et al., PLos Pathog. 2017).
wt39 and wt51 cells were established from JET cells infected with HTLV-1 molecular clone using limiting dilution (Matsuo M. et al., Nature communications. 2021).
HTLV-1 molecular clone, J-Lat cells (9.2 and 10.6) were obtained from the National Institutes of Health AIDs reagent Program.
TCR deficient Jurkat cells were kindly gifted by Takamasa Ueno (Kumamoto university)

Authentication

None of the cell lines used were authenticated.

Mycoplasma contamination

All cell lines tested negative for mycoplasma contamination.

Commonly misidentified lines
(See ICLAC register)

None of the cell lines used in this study are listed in this database.

Plants

Seed stocks

n/a

Novel plant genotypes

n/a

Authentication

n/a

ChIP-seq

Data deposition

☒ Confirm that both raw and final processed data have been deposited in a public database such as GEO.

☐ Confirm that you have deposited or provided access to graph files (e.g. BED files) for the called peaks.

Data access links
May remain private before publication.

Data supporting the findings reported in this study are available from the corresponding author upon request. Raw sequence files (fastq) for ChIP-seq(SUB15170571), ATAC-seq and genomic DNA sequences(SUB15174788) have been deposited to SRA under the bioproject: PRJNA1236037.

Files in database submission

Raw fastq files and processed bam files from ChIP-seq data are following:
[Fastq:SRR32706051] [bam:SRR32764928] 21_wt39_RUNX1_ChIP_replicate_01
[Fastq:SRR32706051] [bam:SRR32764927] 23_wt51_RUNX1_ChIP_replicate_01

[Fastq:SRR32706017] [bam:SRR32764916] 25_wt39_CBFb_ChIP_replicate_01
[Fastq:SRR32706013] [bam:SRR32764905] 27_wt51_CBFb_ChIP_replicate_01
[Fastq:SRR32706011] [bam:SRR32764904] 29_wt39_GATA3_ChIP_replicate_01
[Fastq:SRR32706049] [bam:SRR32764903] 31_wt51_GATA3_ChIP_replicate_01
[Fastq:SRR32706047] [bam:SRR32764902] 33_wt39_ETS1_ChIP_replicate_01
[Fastq:SRR32706045] [bam:SRR32764901] 35_wt51_ETS1_ChIP_replicate_01
[Fastq:SRR32706043] [bam:SRR32764900] 37_wt39_HDAC3_ChIP_replicate_01
[Fastq:SRR32706041] [bam:SRR32764899] 39_wt51_HDAC3_ChIP_replicate_01
[Fastq:SRR32706038] [bam:SRR32764926] 41_wt39_Sin3A_ChIP_replicate_01
[Fastq:SRR32706036] [bam:SRR32764925] 42_wt51_Sin3A_ChIP_replicate_01
[Fastq:SRR32706034] [bam:SRR32764924] 43_AI-5_RUNX1_ChIP_replicate_01
[Fastq:SRR32706033] [bam:SRR32764923] 44_AI-9_RUNX1_ChIP_replicate_01
[Fastq:SRR32706032] [bam:SRR32764922] 45_AI-5_CBFb_ChIP_replicate_01
[Fastq:SRR32706031] [bam:SRR32764921] 46_AI-9_CBFb_ChIP_replicate_01
[Fastq:SRR32706030] [bam:SRR32764920] 47_AI-5_GATA3_ChIP_replicate_01
[Fastq:SRR32706029] [bam:SRR32764919] 48_AI-9_GATA3_ChIP_replicate_01
[Fastq:SRR32706027] [bam:SRR32764918] 49_AI-5_ETS1_ChIP_replicate_01
[Fastq:SRR32706026] [bam:SRR32764917] 50_AI-9_ETS1_ChIP_replicate_01
[Fastq:SRR32706025] [bam:SRR32764915] 51_AI-5_HDAC3_ChIP_replicate_01
[Fastq:SRR32706024] [bam:SRR32764914] 52_AI-9_HDAC3_ChIP_replicate_01
[Fastq:SRR32706023] [bam:SRR32764913] 53_AI-5_Sin3A_ChIP_replicate_01
[Fastq:SRR32706022] [bam:SRR32764912] 54_AI-9_Sin3A_ChIP_replicate_01
[Fastq:SRR32706021] [bam:SRR32764911] 55_JEX_wt_bulk_RUNX1_ChIP_replicate_01
[Fastq:SRR32706020] [bam:SRR32764910] 56_JEX_s-mut_bulk_RUNX1_ChIP_replicate_01
[Fastq:SRR32706019] [bam:SRR32764909] 57_JEX_wt_bulk_CBFb_ChIP_replicate_01
[Fastq:SRR32706018] [bam:SRR32764908] 58_JEX_s-mut_bulk_CBFb_ChIP_replicate_01
[Fastq:SRR32706016] [bam:SRR32764907] 59_JEX_wt_bulk_HDAC3_ChIP_replicate_01
[Fastq:SRR32706015] [bam:SRR32764906] 60_JEX_s-mut_bulk_HDAC3_ChIP_replicate_01
[Fastq:SRR32706050] 22_wt39_RUNX1_ChIP_replicate_02
[Fastq:SRR32706028] 24_wt51_RUNX1_ChIP_replicate_02
[Fastq:SRR32706014] 26_wt39_CBFb_ChIP_replicate_02
[Fastq:SRR32706012] 28_wt51_CBFb_ChIP_replicate_02
[Fastq:SRR32706010] 30_wt39_GATA3_ChIP_replicate_02
[Fastq:SRR32706048] 32_wt51_GATA3_ChIP_replicate_02
[Fastq:SRR32706046] 34_wt39_ETS1_ChIP_replicate_02
[Fastq:SRR32706044] 36_wt51_ETS1_ChIP_replicate_02
[Fastq:SRR32706042] 38_wt39_HDAC3_ChIP_replicate_02
[Fastq:SRR32706040] 40_wt51_HDAC3_ChIP_replicate_02
[Fastq:SRR32706037] 61_wt39_Sin3A_ChIP_replicate_02
[Fastq:SRR32706035] 62_wt51_Sin3A_ChIP_replicate_02

Genome browser session
(e.g. UCSC)

Provide a link to an anonymized genome browser session for "Initial submission" and "Revised version" documents only, to enable peer review. Write "no longer applicable" for "Final submission" documents.

Methodology

Replicates

All results using cell line sample shown in the manuscript were validated by duplicate.
ChIP-seq data using patients samples were performed by single assay because of the limitation with few cell number.

Sequencing depth

In samples with DNA-capture, libraries were sequenced by Illumina MiSeq to obtain paired-end reads around 1 million reads using the following read length: read1_75 bp; read2_75 bp.

Antibodies

anti-RUNX1 (abcam ab23980), anti-CBFb (abcam ab195411), anti-GATA3 (CST 5852P), anti-HDAC3 (Invitrogen PA5-85378), anti-ETS1 (CST 14069S) and anti Sin3A (CST 7691); 1:250 dilution for conventional ChIP. For Cut and Run, the antibodies are used in 1:50 dilution.

Peak calling parameters

No peak calling was performed.

Data quality

The quality of raw data fastq files were determined with FastQC and the reads having phred score >20 were filtered for mapping.

Software

 FastQC (version 0.10.0) – Checking quality of fastq file (https://www.bioinformatics.babraham.ac.uk/projects/fastqc/)
 cutadapt (version 1.18) – Removing the adapter sequences (https://cutadapt.readthedocs.io/en/stable/index.html)
 PRINSEQ (version 0.20.4) – Cleaning the sequence data (http://prinseq.sourceforge.net/)
 BWA (version 0.7.12) – Alignment for DNA-seq, MNase-seq, ChIP-seq (https://sourceforge.net/projects/bio-bwa)
 SAMTools (version 1.11) – Operating sam and bam files (http://www.htslib.org/download/)
 picard (version 2.0.1) – PCR replicate removal (https://broadinstitute.github.io/picard/)
 IGV (version 2.8.0) – Used to visualize mRNA-seq, MNase-seq and ChIP-seq data (https://software.broadinstitute.org/software/igv/download)

Flow Cytometry

Plots

Confirm that:

☒ The axis labels state the marker and fluorochrome used (e.g. CD4-FITC).

☒ The axis scales are clearly visible. Include numbers along axes only for bottom left plot of group (a 'group' is an analysis of identical markers).

☒ All plots are contour plots with outliers or pseudocolor plots.

☒ A numerical value for number of cells or percentage (with statistics) is provided.

Methodology

Sample preparation	shRNA-RUNX1-transduced Jurkat cells were stained by PE-labeled anti-RUNX1 antibody (RXDMC, Invitrogen). JET cell clones (wt39 and wt51) infected with HTLV-1 molecular clone, J-Lat cells (9.2 and 10.6) and PBMCs from ACs and ATL patients were treated with Ro5-3335 for 24 hours. Enhanced 5'LTR reactivation in the JET cell clones and J-Lat cells was measured by expression of tdTomato and GFP, respectively. Ro5-3335-treated PBMCs were stained with PerCP/Cy5.5-labeled anti-CD4 mAb (OKT4, BioLegend) and FITC-labeled anti-Tax mAb. RUNX1-transduced TBX4B and MT-1 cells were stained with FITC-labeled anti-Tax mAb (Lt4) for 30 min at room temperature. rHIV-infected Jurkat and Molt4 cells were detected upon staining with LIVE/DEAD Fixable Near-IR Cell Stain Kit (Thermo Fisher Scientific) and FITC-labeled anti-HIV p24 mAb (Beckman). PBMCs from three ATL patients were stained with anti CADM1 mAb and secondary antibody (Alexa Fluor647-labeled Goat Anti-Chicken IgY H&L ab) and BV421-labeled anti CD7 mAb (M-T701; BD Biosciences), APC-labeled anti CD45RO mAb (UCHL1; Biolegend), and FITC-labeled anti-Tax mAb (Lt4).
Instrument	Processed cells were detected by flow cytometry using BD FACSVerse and Sony Biotechnology SH800S Cell Sorter
Software	Flow cytometry data was analyzed using FlowJo (version 9.9.6).
Cell population abundance	not applicable
Gating strategy	Primary viable cells were gated by forward scatter (FSC) vs. side scatter (SSC). After gating, deadcells were stained by LIVE/DEAD Fixable Near-IR Dead Cell Stain Kit (Thermo Fisher Scientific) and then excluded as the positive cells.

☒ Tick this box to confirm that a figure exemplifying the gating strategy is provided in the Supplementary Information.
