# Supplementary material for: Intragenic viral silencer element regulates HTLV-1 latency via RUNX complex recruitment
Source: Nat Microbiol. 2025 May 13;10(6):1447–62. doi: 10.1038/s41564-025-02006-7 (PMC12137137; doi:10.1038/s41564-025-02006-7)
Supplement: Supplementary file 8 — Flow cytometry gating strategy and unprocessed western blot source data. [file 41564_2025_2006_MOESM8_ESM.pdf]

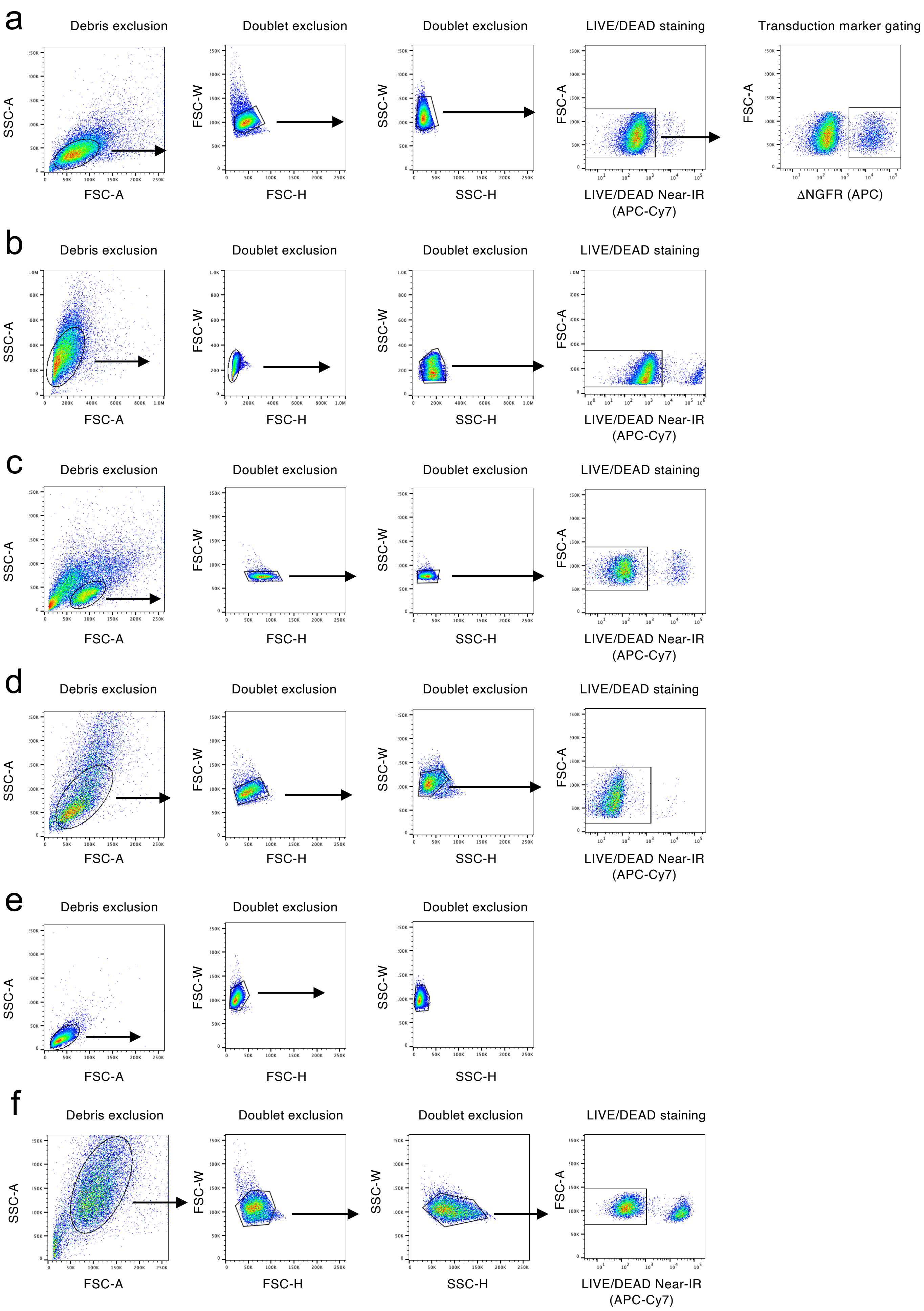
a
Debris exclusion
Doublet exclusion
Doublet exclusion
LIVE/DEAD staining
Transduction marker gating
SSC-A
FSC-A
FSC-W
FSC-H
SSC-W
SSC-H
FSC-A
LIVE/DEAD Near-IR (APC-Cy7)
FSC-A
ΔNGFR (APC)
b
Debris exclusion
Doublet exclusion
Doublet exclusion
LIVE/DEAD staining
SSC-A
FSC-A
FSC-W
FSC-H
SSC-W
SSC-H
FSC-A
LIVE/DEAD Near-IR (APC-Cy7)
c
Debris exclusion
Doublet exclusion
Doublet exclusion
LIVE/DEAD staining
SSC-A
FSC-A
FSC-W
FSC-H
SSC-W
SSC-H
FSC-A
LIVE/DEAD Near-IR (APC-Cy7)
d
Debris exclusion
Doublet exclusion
Doublet exclusion
LIVE/DEAD staining
SSC-A
FSC-A
FSC-W
FSC-H
SSC-W
SSC-H
FSC-A
LIVE/DEAD Near-IR (APC-Cy7)
e
Debris exclusion
Doublet exclusion
Doublet exclusion
SSC-A
FSC-A
FSC-W
FSC-H
SSC-W
SSC-H
f
Debris exclusion
Doublet exclusion
Doublet exclusion
LIVE/DEAD staining
SSC-A
FSC-A
FSC-W
FSC-H
SSC-W
SSC-H
FSC-A
LIVE/DEAD Near-IR (APC-Cy7)

g

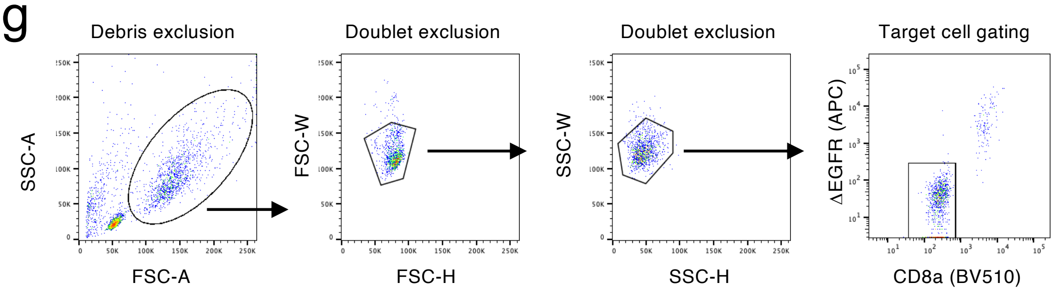


h

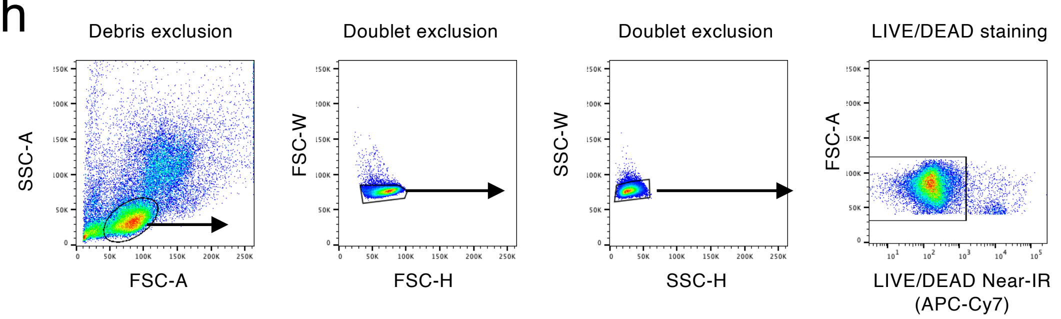


**Gating strategy for Fig.2h, Fig.3g/Ext fig.6bc, Fig.3h/Ext fig.6d, Fig.5e, Fig.5g, Ext fig.4c, Ext fig.6g, Ext fig.7cd.**

**a**, Flow-cytometry gating strategy for fig. 2h. The Near-IR LIVE/DEAD non-stained, live cell population were gated after doublet removal by FSC/SSC. Empty/RUNX1- ΔNGFR transduced cells were gated for quantifying the Tax$^{+}$ cells (%) shown in fig.2h.

**b**, Flow-cytometry gating strategy for fig.3g/Ext data fig.6bc. The Near-IR LIVE/DEAD non-stained, live cell population were gated after doublet removal by FSC/SSC. The percentage of tdTomato positive cells, shown in fig.3g/Ext data fig.6bc were calculated using the live cell population.

**c**, Flow-cytometry gating strategy for fig.3h/Ext data fig.6d. Living CD4 T cells (%) were calculated using the LIVE/DEAD non-stained, live cell population gated after doublet removal by FSC/SSC and total number of input cells. The percentage of Tax positive cells, shown in Ext data fig.6d were calculated using the living CD4 T cells and Tax$^{+}$ cells.

**d**, Flow-cytometry gating strategy for fig.5e. The Near-IR LIVE/DEAD non-stained, live cell population were gated after doublet removal by FSC/SSC. HIV-1 p24+ cells shown in fig.5e were calculated using the live cell population.

**e**, Flow-cytometry gating strategy for fig.5g. The cells for visualizing LIVE/DEAD (APC-Cy7) and HIV-1 p24 (FITC) in fig.5e were gated after doublet exclusion by FSC/SSC.

**f**, Flow-cytometry gating strategy for Ext. Data Fig. 4cd The Near-IR LIVE/DEAD non-stained, live cell population were gated after doublet removal by FSC/SSC. shRNA transduced cells were gated for quantifying the RUNX1 MFI as shown in for Ext. Data Fig. 4cd.

**g**, Flow-cytometry gating strategy for Ext data fig.6g. The CD8a(-), ΔEGFR(-) target cells shown in Ext data fig. 6g were gated after doublet exclusion by FSC/SSC.

**h**, Flow-cytometry gating strategy for Ext data fig.7cd. The plots shown in Ext data fig.7cd were created using the LIVE/DEAD non-stained, live cell population gated after doublet removal by FSC/SSC.

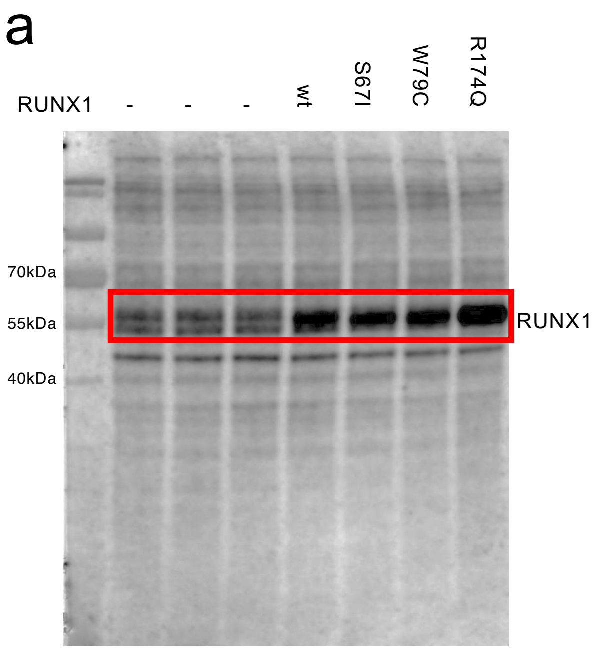


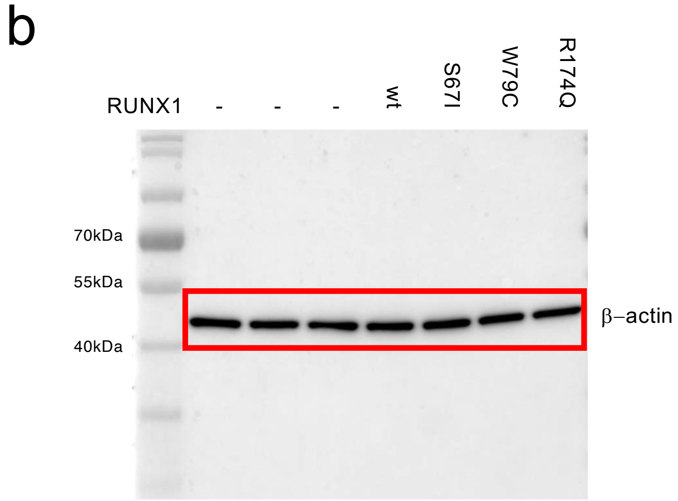


**Unprocessed blots for fig.2f**

**a**, RUNX1 and mutants overexpressed in 293T cells

**b,** β-actin for internal control
